# (*C-meso-N-meso*-5,12-Dimethyl-7,14-diphenyl-1,4,8,11-tetra­aza­cyclo­tetra­deca-4,11-diene)nickel(II) bis­[*O*,*O*′-bis­(4-methyl­phen­yl) dithio­phosphate]

**DOI:** 10.1107/S160053681004849X

**Published:** 2010-11-27

**Authors:** Li-Ke Zou, Bin Xie, Jian-Shen Feng, Chuan Lai

**Affiliations:** a College of Chemistry and Pharmaceutical Engineering, Sichuan University of Science and Engineering, 643000 Zigong, Sichuan, People’s Republic of China

## Abstract

The title complex, [Ni(C_24_H_32_N_4_)](C_14_H_14_O_2_PS_2_)_2_, comprises a centrosymmetric [Ni(*meso*-diphen­yl[14]dien)]^2+^ dication (*meso*-diphen­yl[14]dien is *C-meso-N-meso*-5,12-dimethyl-7,14-diphenyl-1,4,8,11-tetra­aza­cyclo­tetra­deca-4,11-diene) and two *O*,*O*′-bis­(4-methyl­phen­yl) dithio­phosphate anions. The Ni^II^ ion lies on an inversion center and is chelated by a tetra­amine macrocycle ligand in a slightly distorted NiN_4_ square-planar geometry. Two S atoms from symmetry-related anions are located in pseudo-axial positions with respect to the Ni^II^ ion, with Ni⋯S distances of 3.1869 (8) Å. In the crystal, bifurcated inter­molecular N—H⋯S(S) hydrogen bonds connect cations and pairs of anions into three-component clusters. Weak inter­molecular C—H⋯S hydrogen bonds link these clusters into chains along [100].

## Related literature

For the synthesis of the tetra­mine macrocyclic ligand, see: Curtis (2001) ▶. For general background to tetra­mine macrocycles, see: Aoki & Kimura (2002 ▶); For transition metal complexes with *O*,*O*′-dialkyl­dithio­phosphate ligands, see: Drew *et al.* (1987 ▶); Liaw *et al.* (2005 ▶); Zou *et al.* (2009 ▶). For the synthesis and crystal structures of related macrocyclic nickel and copper complexes, see: Feng *et al.* (2009 ▶); He *et al.* (2010 ▶); Xie *et al.* (2009 ▶). For standard bond-length data, see: Allen *et al.* (1987 ▶).
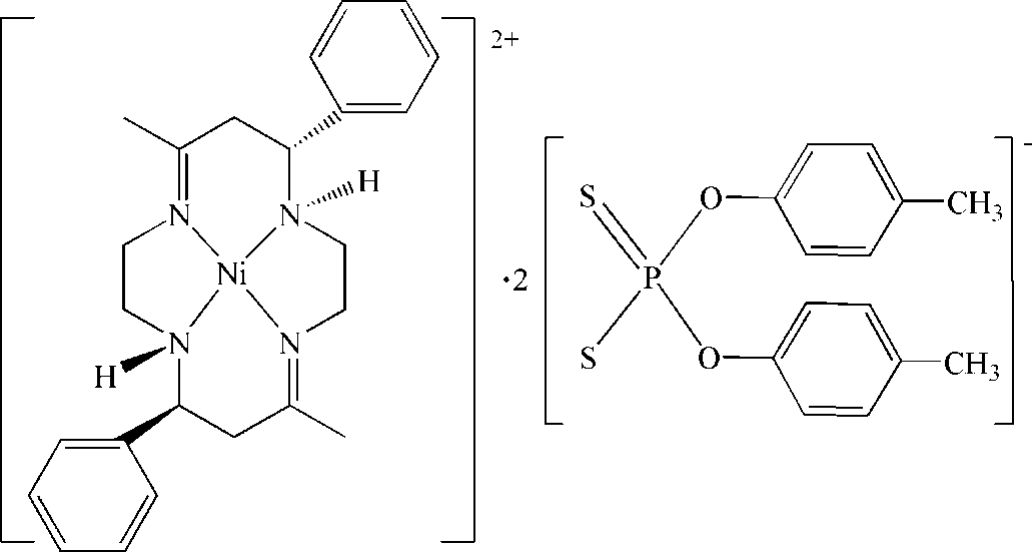


## Experimental

### 

#### Crystal data

[Ni(C_24_H_32_N_4_)](C_14_H_14_O_2_PS_2_)_2_
*M* *_r_* = 1053.93
Monoclinic, 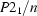

*a* = 10.04828 (18) Å
*b* = 19.6896 (4) Å
*c* = 13.5112 (3) Å
β = 106.900 (2)°
*V* = 2557.69 (9) Å^3^
*Z* = 2
Mo *K*α radiation
μ = 0.65 mm^−1^
*T* = 150 K
0.22 × 0.18 × 0.16 mm

#### Data collection

Oxford Diffraction Xcalibur Eos diffractometer
Absorption correction: multi-scan (*CrysAlis PRO RED*; Oxford Diffraction, 2009 ▶) *T*_min_ = 0.930, *T*_max_ = 1
11501 measured reflections
5232 independent reflections
4326 reflections with *I* > 2σ(*I*)
*R*_int_ = 0.025

#### Refinement

*R*[*F* ^2^ > 2σ(*F* ^2^)] = 0.036
*wR*(*F* ^2^) = 0.085
*S* = 1.01
5232 reflections
307 parameters
H-atom parameters constrained
Δρ_max_ = 0.39 e Å^−3^
Δρ_min_ = −0.28 e Å^−3^

### 

Data collection: *CrysAlis PRO CCD* (Oxford Diffraction, 2009 ▶); cell refinement: *CrysAlis PRO CCD*; data reduction: *CrysAlis PRO RED* (Oxford Diffraction, 2009 ▶); program(s) used to solve structure: *SHELXS97* (Sheldrick, 2008 ▶); program(s) used to refine structure: *SHELXL97* (Sheldrick, 2008 ▶); molecular graphics: *ORTEP-3 for Windows* (Farrugia, 1997 ▶); software used to prepare material for publication: *SHELXL97.*

## Supplementary Material

Crystal structure: contains datablocks I, global. DOI: 10.1107/S160053681004849X/lh5160sup1.cif

Structure factors: contains datablocks I. DOI: 10.1107/S160053681004849X/lh5160Isup2.hkl

Additional supplementary materials:  crystallographic information; 3D view; checkCIF report

## Figures and Tables

**Table 1:** Hydrogen-bond geometry (Å, °)

*D*—H⋯*A*	*D*—H	H⋯*A*	*D*⋯*A*	*D*—H⋯*A*
N2—H2⋯S1	0.93	2.87	3.4652 (17)	123
N2—H2⋯S2	0.93	2.71	3.5789 (17)	156
C5—H5*B*⋯S2^i^	0.98	2.87	3.815 (2)	162

Symmetry code: (i) 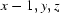.

**Fig. 1.**
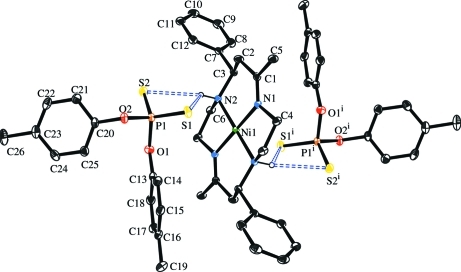
The molecular structure of the title complex, showing the atom-numbering scheme with displacement ellipsoids at 30% probability level. H atoms on N are represented as small spheres of arbitary radii and H atoms on C have been omitted for the sake of clarity. Hydrogen-bonds are shown as dashed lines. [Symmetry code: (i) -x + 1, -y, -z + 1].

## supplementary crystallographic information

### Comment

The significance of synthetic tetramine macrocycles is most obvious because of their strong chelating ability and analogy to naturally occurring macrocyclic systems, therefore the synthesis and potential use of their transition-metal complexes have been extensively studied (Aoki *et al.*, 2002). At the same time, the transition-metal complexes of *O*,*O*'- dialkyldithiophosphate ligands (DDP) have attracted our attention due to their luxuriant variety of coordination bonding characteristics (Drew *et al.*, 1987; Liaw *et al.*, 2005) and potential application as mimetic hydrolases for carboxylic acid esters (Zou *et al.*, 2009). For these reasons, we have recently reported several structures of tetramine macrocyclic transition-metal adducts with *O*,*O*'-dialkyldithiophosphate (Feng *et al.*, 2009; Xie *et al.*, 2009; He *et al.*, 2010). Herein, we report the structure of [Ni(*meso*-diphenyl[14]dien)][S_2_P(OC_6_H_4_Me-4)_2_]_2_, where *meso*-diphenyl[14]dien is *C-meso-N-meso*-5,12-dimethyl-7,14- diphenyl-1,4,8,11-tetraazacyclotetradeca-4,11-diene.

The molecular of the title complex comprises a complex mononuclear [Ni(*meso*-diphenyl[14]dien)]^2+^ cation and two *O*,*O*'-bis(4-methylphenyl) dithiophosphate anions. The Ni^II^ atom lies on an inversion centre and is chelated by four N atoms from the macrocyclic tetramine *meso*-diphenyl[14]dien in a slightly distorted NiN_4_ square-planar geometry (Fig.1). Two uncoordinated *O*,*O*'-bis(4-methylphenyl) dithiophosphate anions occupy pseudo-axial positions with Cu···S distances of 3.1869 (7) Å, forming a octahedral type arrangement. Intermolecular N—H···S and C—H···S hydrogen bonds are present between the anions and the cations. All bond lengths (Allen *et al.*, 1987) and angles in the complex are within normal ranges.

### Experimental

The tetraamine macrocyclic ligand 5,12-Dimethyl-7,14-diphenyl-1,4,8,11- tetraazacyclotetradeca-4,11-diene (diphenyl[14]dien) and [Ni(diphenyl[14]dien)](ClO_4_)_2_ (a mixture of *meso*- and *rac*- isomers) were prepared according to the procedure described by Curtis (2001).

The title complex was prepared by a modified method according to our previous work (Xie *et al.*, 2009). A cellulose thimble containing 0.634 g (1 mmol) of mixed isomers of [Ni(diphenyl[14]dien)](ClO_4_)_2_ was placed in a Soxhlet apparatus. The isomers were slowly extracted to a solution of 0.767 g (2 mmol) [(C_2_H_5_)_2_NH_2_][S_2_P(OC_6_H_4_Me-4)_2_] in 70 mL methanol (extraction solvent) and the less soluble *meso*-isomer of the adduct, [Ni(diphenyl[14]dien)][S_2_P(OC_6_H_4_Me-4)_2_]_2_, was slowly pricipitated out during the extraction procedure. The whole extraction process lasted about 36 hours and then refluxed for another 4 hours. After cooling to room temperature, the solid was filtered off and washed successively with methanol, acetone and diethyl ether. The crude product was dissolved in hot dimethylformamide and filtered, the filtrate was kept at room temperature and pale-violet block crystals suitable for X-ray diffraction studies were obtained after three months.

### Refinement

H atoms attached to C and N atoms were fixed geometrically and treated as riding, with C—H = 1.00Å (methine), 0.99Å (methylene), 0.98Å (methyl), 0.95Å (aromatic) and N—H = 0.93 Å. The *U*_iso_(H) = 1.5*U*_eq_(C) for methyl groups and *U*_iso_(H) = 1.2*U*_eq_(C, N) for all other carbon and nitrogen bound H atoms.

### Crystal data

**Table:**

[Ni(C_24_H_32_N_4_)](C_14_H_14_O_2_PS_2_)_2_	*F*(000) = 1108
*M**_r_* = 1053.93	*D*_x_ = 1.368 Mg m^−^^3^
Monoclinic, *P*2_1_/*n*	Mo *K*α radiation, λ = 0.7107 Å
Hall symbol: -P 2yn	Cell parameters from 5452 reflections
*a* = 10.04828 (18) Å	θ = 3.0–29.2°
*b* = 19.6896 (4) Å	µ = 0.65 mm^−^^1^
*c* = 13.5112 (3) Å	*T* = 150 K
β = 106.900 (2)°	Block, pale-violet
*V* = 2557.69 (9) Å^3^	0.22 × 0.18 × 0.16 mm
*Z* = 2	

### Data collection

**Table:**

Oxford Diffraction Xcalibur Eos diffractometer	5232 independent reflections
Radiation source: fine-focus sealed tube	4326 reflections with *I* > 2σ(*I*)
graphite	*R*_int_ = 0.025
Detector resolution: 16.0874 pixels mm^-1^	θ_max_ = 26.4°, θ_min_ = 3.0°
ω scans	*h* = −12→12
Absorption correction: multi-scan (*CrysAlis PRO* RED; Oxford Diffraction, 2009)	*k* = −24→22
*T*_min_ = 0.930, *T*_max_ = 1.0	*l* = −16→16
11501 measured reflections	

### Refinement

**Table:**

Refinement on *F*^2^	Primary atom site location: structure-invariant direct methods
Least-squares matrix: full	Secondary atom site location: difference Fourier map
*R*[*F*^2^ > 2σ(*F*^2^)] = 0.036	Hydrogen site location: inferred from neighbouring sites
*wR*(*F*^2^) = 0.085	H-atom parameters constrained
*S* = 1.01	*w* = 1/[σ^2^(*F*_o_^2^) + (0.033*P*)^2^ + 1.139*P*] where *P* = (*F*_o_^2^ + 2*F*_c_^2^)/3
5232 reflections	(Δ/σ)_max_ = 0.001
307 parameters	Δρ_max_ = 0.39 e Å^−^^3^
0 restraints	Δρ_min_ = −0.28 e Å^−^^3^

### Special details

**Table:**

Geometry. All esds (except the esd in the dihedral angle between two l.s. planes) are estimated using the full covariance matrix. The cell esds are taken into account individually in the estimation of esds in distances, angles and torsion angles; correlations between esds in cell parameters are only used when they are defined by crystal symmetry. An approximate (isotropic) treatment of cell esds is used for estimating esds involving l.s. planes.
Refinement. Refinement of F^2^ against ALL reflections. The weighted R-factor wR and goodness of fit S are based on F^2^, conventional R-factors R are based on F, with F set to zero for negative F^2^. The threshold expression of F^2^ > 2sigma(F^2^) is used only for calculating R-factors(gt) etc. and is not relevant to the choice of reflections for refinement. R-factors based on F^2^ are statistically about twice as large as those based on F, and R- factors based on ALL data will be even larger.

### Fractional atomic coordinates and isotropic or equivalent isotropic displacement parameters (Å^2^)

**Table:**

	*x*	*y*	*z*	*U*_iso_*/*U*_eq_	
Ni1	0.5000	0.0000	0.5000	0.01821 (10)	
N2	0.65018 (15)	0.05440 (8)	0.58612 (13)	0.0187 (4)	
H2	0.6883	0.0785	0.5417	0.022*	
N1	0.36007 (16)	0.06810 (8)	0.49274 (12)	0.0194 (4)	
C7	0.7393 (2)	0.14121 (10)	0.72455 (16)	0.0231 (4)	
C3	0.6128 (2)	0.10500 (10)	0.65498 (16)	0.0230 (4)	
H3	0.5655	0.0804	0.7001	0.028*	
C12	0.8221 (2)	0.18311 (11)	0.68330 (17)	0.0273 (5)	
H12	0.8003	0.1886	0.6105	0.033*	
C1	0.37339 (19)	0.12907 (10)	0.52677 (16)	0.0215 (4)	
C4	0.22025 (19)	0.04534 (10)	0.42970 (17)	0.0257 (5)	
H4A	0.1666	0.0842	0.3917	0.031*	
H4B	0.1683	0.0256	0.4748	0.031*	
C6	0.7588 (2)	0.00694 (10)	0.64466 (16)	0.0248 (5)	
H6A	0.7291	−0.0150	0.7007	0.030*	
H6B	0.8467	0.0317	0.6761	0.030*	
C2	0.5110 (2)	0.15594 (11)	0.59068 (18)	0.0301 (5)	
H2B	0.4934	0.1910	0.6379	0.036*	
H2A	0.5562	0.1788	0.5437	0.036*	
C5	0.2573 (2)	0.18000 (11)	0.50298 (18)	0.0294 (5)	
H5A	0.2449	0.1989	0.4338	0.044*	
H5C	0.2801	0.2167	0.5542	0.044*	
H5B	0.1711	0.1577	0.5054	0.044*	
C11	0.9352 (2)	0.21660 (12)	0.74757 (18)	0.0330 (5)	
H11	0.9908	0.2450	0.7187	0.040*	
C9	0.8874 (2)	0.16789 (13)	0.89515 (18)	0.0382 (6)	
H9	0.9094	0.1626	0.9680	0.046*	
C8	0.7738 (2)	0.13401 (12)	0.83067 (17)	0.0310 (5)	
H8	0.7190	0.1055	0.8600	0.037*	
C10	0.9680 (2)	0.20904 (12)	0.85370 (19)	0.0368 (6)	
H10	1.0459	0.2322	0.8977	0.044*	
P1	0.73840 (5)	0.08481 (3)	0.33089 (4)	0.02337 (13)	
S1	0.54518 (6)	0.10019 (3)	0.32732 (5)	0.03221 (15)	
S2	0.88615 (6)	0.11448 (3)	0.45192 (4)	0.03143 (14)	
O2	0.75506 (14)	0.11660 (7)	0.22395 (11)	0.0274 (3)	
O1	0.77128 (15)	0.00483 (7)	0.31873 (11)	0.0272 (3)	
C20	0.8809 (2)	0.11224 (11)	0.19887 (16)	0.0255 (5)	
C23	1.1212 (2)	0.10828 (13)	0.13572 (17)	0.0332 (6)	
C14	0.5849 (2)	−0.02229 (11)	0.16048 (17)	0.0286 (5)	
H14	0.5515	0.0231	0.1527	0.034*	
C13	0.6975 (2)	−0.04031 (11)	0.24365 (17)	0.0261 (5)	
C25	0.9101 (2)	0.05523 (12)	0.14963 (17)	0.0306 (5)	
H25	0.8484	0.0175	0.1366	0.037*	
C17	0.6815 (3)	−0.15446 (12)	0.1808 (2)	0.0385 (6)	
H17	0.7156	−0.1997	0.1883	0.046*	
C21	0.9712 (2)	0.16664 (11)	0.21960 (17)	0.0304 (5)	
H21	0.9519	0.2053	0.2552	0.037*	
C16	0.5682 (2)	−0.13849 (12)	0.09778 (19)	0.0349 (6)	
C15	0.5220 (2)	−0.07171 (12)	0.08889 (18)	0.0314 (5)	
H15	0.4450	−0.0594	0.0320	0.038*	
C18	0.7466 (2)	−0.10621 (11)	0.25325 (19)	0.0324 (5)	
H18	0.8247	−0.1184	0.3094	0.039*	
C22	1.0910 (2)	0.16418 (13)	0.18768 (17)	0.0342 (6)	
H22	1.1533	0.2016	0.2018	0.041*	
C26	1.2453 (2)	0.10744 (16)	0.0941 (2)	0.0518 (8)	
H26B	1.2785	0.0607	0.0935	0.078*	
H26C	1.3196	0.1355	0.1382	0.078*	
H26A	1.2182	0.1256	0.0235	0.078*	
C24	1.0306 (2)	0.05370 (13)	0.11934 (17)	0.0341 (5)	
H24	1.0516	0.0141	0.0865	0.041*	
C19	0.4963 (3)	−0.19066 (14)	0.0184 (2)	0.0526 (8)	
H19A	0.4047	−0.2011	0.0265	0.079*	
H19C	0.5525	−0.2322	0.0282	0.079*	
H19B	0.4851	−0.1726	−0.0512	0.079*	

### Atomic displacement parameters (Å^2^)

**Table:**

	*U*^11^	*U*^22^	*U*^33^	*U*^12^	*U*^13^	*U*^23^
Ni1	0.01354 (17)	0.01832 (19)	0.0208 (2)	0.00210 (14)	0.00195 (14)	−0.00173 (15)
N2	0.0153 (8)	0.0185 (8)	0.0218 (9)	0.0012 (7)	0.0042 (7)	0.0012 (7)
N1	0.0160 (8)	0.0202 (9)	0.0208 (9)	0.0003 (7)	0.0037 (7)	0.0022 (7)
C7	0.0198 (10)	0.0246 (11)	0.0238 (11)	0.0035 (8)	0.0043 (8)	−0.0058 (9)
C3	0.0199 (10)	0.0281 (11)	0.0219 (11)	−0.0018 (9)	0.0074 (8)	−0.0063 (9)
C12	0.0248 (10)	0.0302 (12)	0.0257 (12)	0.0012 (9)	0.0052 (9)	−0.0035 (10)
C1	0.0192 (10)	0.0217 (11)	0.0238 (11)	0.0028 (8)	0.0068 (8)	0.0009 (9)
C4	0.0142 (9)	0.0213 (11)	0.0369 (13)	0.0017 (8)	−0.0001 (9)	0.0005 (10)
C6	0.0201 (10)	0.0213 (11)	0.0267 (12)	0.0029 (8)	−0.0030 (8)	0.0037 (9)
C2	0.0204 (10)	0.0256 (12)	0.0405 (14)	0.0028 (9)	0.0029 (9)	−0.0102 (10)
C5	0.0230 (10)	0.0204 (11)	0.0416 (14)	0.0046 (9)	0.0044 (9)	−0.0028 (10)
C11	0.0265 (11)	0.0286 (13)	0.0429 (15)	−0.0019 (10)	0.0088 (10)	−0.0077 (11)
C9	0.0380 (13)	0.0442 (15)	0.0259 (13)	0.0053 (11)	−0.0009 (10)	−0.0084 (11)
C8	0.0302 (12)	0.0359 (13)	0.0263 (12)	0.0012 (10)	0.0072 (9)	−0.0027 (10)
C10	0.0262 (11)	0.0359 (13)	0.0399 (15)	0.0000 (10)	−0.0036 (10)	−0.0142 (12)
P1	0.0218 (3)	0.0267 (3)	0.0235 (3)	0.0008 (2)	0.0095 (2)	0.0013 (2)
S1	0.0223 (3)	0.0446 (4)	0.0323 (3)	0.0051 (2)	0.0119 (2)	0.0032 (3)
S2	0.0263 (3)	0.0397 (3)	0.0285 (3)	−0.0055 (2)	0.0084 (2)	−0.0055 (3)
O2	0.0245 (7)	0.0321 (8)	0.0281 (8)	0.0041 (6)	0.0117 (6)	0.0071 (7)
O1	0.0287 (8)	0.0258 (8)	0.0256 (8)	0.0026 (6)	0.0055 (6)	0.0008 (7)
C20	0.0226 (10)	0.0339 (12)	0.0215 (11)	0.0019 (9)	0.0088 (8)	0.0068 (10)
C23	0.0220 (11)	0.0558 (16)	0.0203 (11)	0.0021 (11)	0.0037 (9)	0.0110 (11)
C14	0.0326 (12)	0.0261 (11)	0.0283 (12)	−0.0017 (10)	0.0109 (10)	0.0045 (10)
C13	0.0288 (11)	0.0278 (12)	0.0255 (12)	−0.0030 (9)	0.0138 (9)	0.0012 (10)
C25	0.0308 (12)	0.0367 (13)	0.0259 (12)	−0.0037 (10)	0.0107 (9)	−0.0001 (10)
C17	0.0433 (14)	0.0233 (12)	0.0577 (17)	−0.0031 (11)	0.0284 (13)	0.0003 (12)
C21	0.0329 (12)	0.0280 (12)	0.0303 (13)	0.0003 (10)	0.0091 (10)	0.0058 (10)
C16	0.0395 (13)	0.0307 (13)	0.0425 (15)	−0.0141 (11)	0.0247 (12)	−0.0050 (11)
C15	0.0316 (12)	0.0356 (13)	0.0286 (12)	−0.0094 (10)	0.0113 (10)	0.0009 (11)
C18	0.0335 (12)	0.0282 (12)	0.0390 (14)	0.0019 (10)	0.0160 (10)	0.0043 (11)
C22	0.0282 (11)	0.0421 (14)	0.0301 (13)	−0.0079 (11)	0.0052 (10)	0.0121 (11)
C26	0.0261 (12)	0.092 (2)	0.0389 (15)	0.0024 (14)	0.0126 (11)	0.0119 (15)
C24	0.0343 (12)	0.0459 (15)	0.0236 (12)	0.0058 (11)	0.0107 (10)	−0.0011 (11)
C19	0.0566 (17)	0.0418 (16)	0.066 (2)	−0.0235 (13)	0.0277 (15)	−0.0154 (14)

### Geometric parameters (Å, °)

**Table:**

Ni1—N2	1.9385 (15)	C10—H10	0.9500
Ni1—N2^i^	1.9385 (15)	P1—S1	1.9517 (7)
Ni1—N1	1.9247 (16)	P1—S2	1.9514 (8)
Ni1—N1^i^	1.9247 (16)	P1—O2	1.6273 (15)
N2—H2	0.9300	P1—O1	1.6271 (15)
N2—C3	1.484 (2)	O2—C20	1.403 (2)
N2—C6	1.479 (2)	O1—C13	1.390 (3)
N1—C1	1.279 (3)	C20—C25	1.379 (3)
N1—C4	1.485 (2)	C20—C21	1.379 (3)
C7—C3	1.521 (3)	C23—C22	1.386 (3)
C7—C12	1.397 (3)	C23—C26	1.510 (3)
C7—C8	1.381 (3)	C23—C24	1.384 (3)
C3—H3	1.0000	C14—H14	0.9500
C3—C2	1.513 (3)	C14—C13	1.389 (3)
C12—H12	0.9500	C14—C15	1.388 (3)
C12—C11	1.381 (3)	C13—C18	1.381 (3)
C1—C2	1.498 (3)	C25—H25	0.9500
C1—C5	1.500 (3)	C25—C24	1.387 (3)
C4—H4A	0.9900	C17—H17	0.9500
C4—H4B	0.9900	C17—C16	1.382 (3)
C4—C6^i^	1.495 (3)	C17—C18	1.385 (3)
C6—C4^i^	1.495 (3)	C21—H21	0.9500
C6—H6A	0.9900	C21—C22	1.393 (3)
C6—H6B	0.9900	C16—C15	1.388 (3)
C2—H2B	0.9900	C16—C19	1.509 (3)
C2—H2A	0.9900	C15—H15	0.9500
C5—H5A	0.9800	C18—H18	0.9500
C5—H5C	0.9800	C22—H22	0.9500
C5—H5B	0.9800	C26—H26B	0.9800
C11—H11	0.9500	C26—H26C	0.9800
C11—C10	1.383 (3)	C26—H26A	0.9800
C9—H9	0.9500	C24—H24	0.9500
C9—C8	1.389 (3)	C19—H19A	0.9800
C9—C10	1.375 (3)	C19—H19C	0.9800
C8—H8	0.9500	C19—H19B	0.9800
			
Ni1—N2—H2	106.8	C8—C7—C12	118.43 (19)
N2^i^—Ni1—N2	180	C8—C9—H9	119.9
N2—C3—C7	112.54 (15)	C10—C11—H11	119.8
N2—C3—H3	108.0	C10—C9—H9	119.9
N2—C3—C2	109.70 (16)	C10—C9—C8	120.1 (2)
N2—C6—C4^i^	107.56 (16)	S2—P1—S1	118.90 (4)
N2—C6—H6A	110.2	O2—P1—S1	106.07 (6)
N2—C6—H6B	110.2	O2—P1—S2	112.29 (6)
N1—Ni1—N2	94.32 (7)	O1—P1—S1	112.17 (6)
N1—Ni1—N2^i^	85.68 (7)	O1—P1—S2	104.30 (6)
N1^i^—Ni1—N2	85.68 (7)	O1—P1—O2	101.90 (8)
N1^i^—Ni1—N2^i^	94.32 (7)	C20—O2—P1	121.56 (13)
N1^i^—Ni1—N1	180	C20—C25—H25	120.4
N1—C1—C2	121.44 (17)	C20—C25—C24	119.1 (2)
N1—C1—C5	123.84 (18)	C20—C21—H21	120.4
N1—C4—H4A	110.2	C20—C21—C22	119.2 (2)
N1—C4—H4B	110.2	C23—C22—C21	121.4 (2)
N1—C4—C6^i^	107.38 (16)	C23—C22—H22	119.3
C7—C3—H3	108.0	C23—C26—H26B	109.5
C7—C12—H12	119.7	C23—C26—H26C	109.5
C7—C8—C9	121.0 (2)	C23—C26—H26A	109.5
C7—C8—H8	119.5	C23—C24—C25	121.8 (2)
C3—N2—Ni1	116.89 (11)	C23—C24—H24	119.1
C3—N2—H2	106.8	C14—C13—O1	124.28 (19)
C3—C2—H2B	108.1	C14—C15—H15	119.0
C3—C2—H2A	108.1	C13—O1—P1	127.73 (13)
C12—C7—C3	121.19 (18)	C13—C14—H14	120.5
C12—C11—H11	119.8	C13—C18—C17	119.7 (2)
C12—C11—C10	120.3 (2)	C13—C18—H18	120.1
C1—N1—Ni1	129.59 (14)	C25—C20—O2	119.94 (19)
C1—N1—C4	118.19 (16)	C25—C24—H24	119.1
C1—C2—C3	116.97 (17)	C17—C16—C15	117.5 (2)
C1—C2—H2B	108.1	C17—C16—C19	122.2 (2)
C1—C2—H2A	108.1	C17—C18—H18	120.1
C1—C5—H5A	109.5	C21—C20—O2	119.30 (19)
C1—C5—H5C	109.5	C21—C20—C25	120.69 (19)
C1—C5—H5B	109.5	C21—C22—H22	119.3
C4—N1—Ni1	111.99 (12)	C16—C17—H17	119.1
C4^i^—C6—H6A	110.2	C16—C17—C18	121.8 (2)
C4^i^—C6—H6B	110.2	C16—C15—C14	122.1 (2)
H4A—C4—H4B	108.5	C16—C15—H15	119.0
C6—N2—Ni1	107.25 (12)	C16—C19—H19A	109.5
C6—N2—H2	106.8	C16—C19—H19C	109.5
C6—N2—C3	111.77 (15)	C16—C19—H19B	109.5
C6^i^—C4—H4A	110.2	C15—C14—H14	120.5
C6^i^—C4—H4B	110.2	C15—C14—C13	118.9 (2)
H6A—C6—H6B	108.5	C15—C16—C19	120.3 (2)
C2—C3—C7	110.38 (16)	C18—C13—O1	115.63 (19)
C2—C3—H3	108.0	C18—C13—C14	120.0 (2)
C2—C1—C5	114.66 (17)	C18—C17—H17	119.1
H2B—C2—H2A	107.3	C22—C23—C26	121.6 (2)
H5A—C5—H5C	109.5	C22—C21—H21	120.4
H5A—C5—H5B	109.5	H26B—C26—H26C	109.5
H5C—C5—H5B	109.5	H26B—C26—H26A	109.5
C11—C12—C7	120.5 (2)	H26C—C26—H26A	109.5
C11—C12—H12	119.7	C24—C23—C22	117.8 (2)
C11—C10—H10	120.2	C24—C23—C26	120.5 (2)
C9—C8—H8	119.5	C24—C25—H25	120.4
C9—C10—C11	119.7 (2)	H19A—C19—H19C	109.5
C9—C10—H10	120.2	H19A—C19—H19B	109.5
C8—C7—C3	120.38 (19)	H19C—C19—H19B	109.5
			
Ni1—N2—C3—C7	175.86 (13)	C8—C7—C12—C11	−0.2 (3)
Ni1—N2—C3—C2	−60.84 (19)	C8—C9—C10—C11	0.1 (4)
Ni1—N2—C6—C4^i^	45.69 (18)	C10—C9—C8—C7	−0.4 (3)
Ni1—N1—C1—C2	−6.0 (3)	P1—O2—C20—C25	85.7 (2)
Ni1—N1—C1—C5	171.17 (15)	P1—O2—C20—C21	−97.2 (2)
Ni1—N1—C4—C6^i^	−25.2 (2)	P1—O1—C13—C14	4.3 (3)
N2^i^—Ni1—N1—C1	−174.21 (19)	P1—O1—C13—C18	−178.36 (15)
N2—Ni1—N1—C1	5.79 (19)	S1—P1—O2—C20	−177.67 (14)
N2—Ni1—N1—C4	180.00 (13)	S1—P1—O1—C13	50.57 (17)
N2^i^—Ni1—N1—C4	0.00 (13)	S2—P1—O2—C20	50.91 (16)
N2—C3—C2—C1	62.2 (2)	S2—P1—O1—C13	−179.45 (15)
N1—Ni1—N2—C3	28.14 (14)	O2—P1—O1—C13	−62.47 (17)
N1^i^—Ni1—N2—C3	−151.86 (14)	O2—C20—C25—C24	175.78 (19)
N1—Ni1—N2—C6	154.50 (13)	O2—C20—C21—C22	−175.25 (18)
N1^i^—Ni1—N2—C6	−25.50 (13)	O1—P1—O2—C20	−60.14 (16)
N1—C1—C2—C3	−28.3 (3)	O1—C13—C18—C17	−178.68 (19)
C7—C3—C2—C1	−173.27 (18)	C20—C25—C24—C23	−1.1 (3)
C7—C12—C11—C10	0.0 (3)	C20—C21—C22—C23	−0.1 (3)
C3—N2—C6—C4^i^	175.03 (16)	C14—C13—C18—C17	−1.2 (3)
C3—C7—C12—C11	178.85 (18)	C13—C14—C15—C16	0.0 (3)
C3—C7—C8—C9	−178.67 (19)	C25—C20—C21—C22	1.7 (3)
C12—C7—C3—N2	64.4 (2)	C17—C16—C15—C14	−0.7 (3)
C12—C7—C3—C2	−58.5 (2)	C21—C20—C25—C24	−1.2 (3)
C12—C7—C8—C9	0.4 (3)	C16—C17—C18—C13	0.4 (3)
C12—C11—C10—C9	0.1 (3)	C15—C14—C13—O1	178.26 (19)
C1—N1—C4—C6^i^	149.72 (18)	C15—C14—C13—C18	1.0 (3)
C4—N1—C1—C2	−179.86 (19)	C18—C17—C16—C15	0.5 (3)
C4—N1—C1—C5	−2.7 (3)	C18—C17—C16—C19	−180.0 (2)
C6—N2—C3—C7	51.8 (2)	C22—C23—C24—C25	2.6 (3)
C6—N2—C3—C2	175.07 (16)	C26—C23—C22—C21	175.4 (2)
C5—C1—C2—C3	154.36 (19)	C26—C23—C24—C25	−174.9 (2)
C8—C7—C3—N2	−116.5 (2)	C24—C23—C22—C21	−2.1 (3)
C8—C7—C3—C2	120.6 (2)	C19—C16—C15—C14	179.8 (2)

Symmetry codes: (i) −*x*+1, −*y*, −*z*+1.

### Hydrogen-bond geometry (Å, °)

**Table:**

*D*—H···*A*	*D*—H	H···*A*	*D*···*A*	*D*—H···*A*
N2—H2···S1	0.93	2.87	3.4652 (17)	123
N2—H2···S2	0.93	2.71	3.5789 (17)	156
C5—H5B···S2^ii^	0.98	2.87	3.815 (2)	162

Symmetry codes: (ii) *x*−1, *y*, *z*.
